# Transcriptional interference drives intronic polyadenylation at the endogenous *H13/Mcts2* locus

**DOI:** 10.1093/nar/gkag640

**Published:** 2026-06-27

**Authors:** James A Cain, Chuying Yang, Hallgerdur Kolbeinsdottir, Anne-Valerie Gendrel, Hannah E Mischo, Bertille Montibus, Rebecca J Oakey

**Affiliations:** Department of Medical and Molecular Genetics, King’s College London, London SE1 9RT, UK; Department of Medical and Molecular Genetics, King’s College London, London SE1 9RT, UK; Department of Medical and Molecular Genetics, King’s College London, London SE1 9RT, UK; GIMM - Gulbenkian Institute for Molecular Medicine, Lisbon 1649-028, Portugal; Department of Infectious Diseases, King’s College London, London SE1 9RT, UK; Department of Medical and Molecular Genetics, King’s College London, London SE1 9RT, UK; Department of Medical and Molecular Genetics, King’s College London, London SE1 9RT, UK

## Abstract

Over a tenth of mammalian genes are nested within other host genes, raising the question of how both can be co-expressed when concurrent transcription occurs in overlapping genetic space. DNA methylation at nested intragenic CpG island promoters can prevent such conflict by silencing the nested gene. Conversely, when these promoters lack DNA methylation and are active, transcriptional interference is widely assumed to occur, but this has not been mechanistically demonstrated at endogenous mammalian loci. Here, we demonstrate transcriptional interference at an endogenous mammalian host/nested locus, the imprinted *H13/Mcts2* pair. Active nested gene (*Mcts2*) transcription promotes host gene (*H13*) intronic polyadenylation, but when the intragenic *Mcts2* promoter is silenced through DNA methylation, host gene elongation reaches the distal 3′UTR polyadenylation of *H13*. We establish that this intronic polyadenylation depends on the act of transcription itself, independently of DNA methylation at the intragenic *Mcts2* promoter. Moreover, nested gene transcription disrupts host gene elongation even when the upstream intronic polyadenylation signal is genetically ablated. Our findings provide mechanistic insight into the widely held assumption of transcriptional interference and reveal how nested gene transcription can trigger premature termination of host genes, with implications for the hundreds of similarly organised loci across mammalian genomes.

## Graphical Abstract

**Graphical Abstract:**
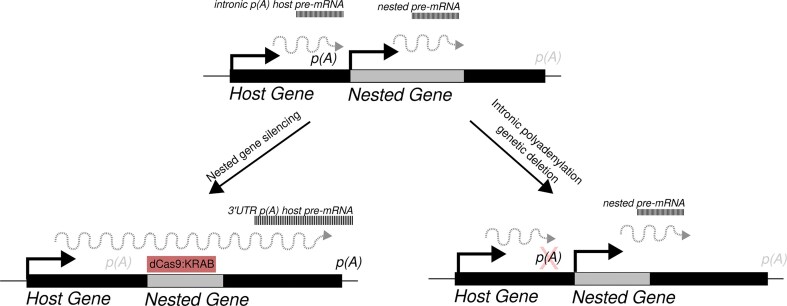

## Introduction

Gene regulation involves a myriad of mechanisms working together, including control of transcription initiation and co-transcriptional processes of splicing and polyadenylation. The resultant fate of RNA transcripts ultimately shapes the functional landscape of the cell [1–3]. An additional layer of complexity arises when these processes intersect at regions of genic overlap, where transcription co-occurs in the same genomic location [4, 5].

*In-vitro* studies examining RNA polymerase II interactions during genic overlap have demonstrated that both antisense or convergent transcription can lead to head-to-head ‘collisions’, acting as barriers that may result in premature termination [6–8]. In contrast, same orientation collisions effectively push RNA polymerases forward [9]. *In vivo*, such occurrences are broadly defined as transcriptional interference, with one transcriptional process directly and *in cis* impeding a second transcriptional process [4]. Transcriptional interference events can be broadly categorised into whether the transcriptional processes are 1) initiation interference: an incoming polymerase transcribing over a second gene’s promoter or 2) elongation interference: a currently transcribing polymerase blocking an incoming polymerase’s elongation. The former has been extensively studied in a variety of species ranging from yeast [10, 11], *Drosophila* [12–14], *Arabidopsis* [15], ants [16], and mammals [17], whereas the latter has been less characterised [18]. Furthermore, the mechanistic demonstration of transcriptional interference at endogenous mammalian loci has remained challenging due to the requirement for demonstrating independence from DNA modifications as one of the criteria for *bona fide* transcriptional interference. Resolving this challenge is particularly relevant at host/nested gene pairs, where one gene is completely embedded within another, a configuration that over a tenth of mammalian genes adopt [19]. When such pairs are co-expressed, a host gene could drive initiation interference of the nested gene, or the nested gene could drive elongation interference of the host.

At overlapping genic regions in mammals, the fate of transcriptional collisions is determined by the relative strength of the competing transcriptional events, dictating whether transcription continues or terminates [20]. These transcriptional outcomes are reinforced through chromatin modifications that accompany gene expression. Specifically, transcription elongation deposits H3K36me3 along the gene body, which subsequently recruits DNMT3B to establish DNA methylation [21, 22]. This process silences intragenic transcription through deposition of DNA methylation at CpG island (CGI) promoters, as demonstrated at numerous loci, and is analogous to initiation transcriptional interference [23–25]. The phenotypic relevance of such transcription-mediated DNA methylation is exemplified in an inherited form of alpha-thalassemia. Here, a genetic deletion (*α*−^ZF^ deletion) removes 18.4kb of intervening sequence, placing the antisense *LUC7L* directly adjacent to the alpha-globin gene *HBA2* and resulting in transcriptional silencing and DNA methylation of the *HBA2* CGI promoter [24]. The capacity of *LUC7L* transcription to silence CGI promoters depends on promoter strength. When synthetic *LUC7L* constructs were inserted antisense and upstream of sense *ACTB, UBC*, or *MYOD1-AS*, all CGI promoters were silenced except for *ACTB*, which is the most highly expressed in the cell type investigated [20]. This indicates that promoters possess differential capacities to maintain activity when challenged by incoming transcription. Supporting these experimental findings, the same study demonstrated that intragenic CGI promoters with high RNA polymerase II occupancy resist DNA methylation deposition, and this correlates with lower RNA polymerase II occupancy at upstream CGIs. These observations further establish transcriptional strength as a key determinant in resolving RNA polymerase II collisions.

The consequences of transcriptional collisions influence host 3′ pre-mRNA processing mechanisms, such as polyadenylation, at various endogenous loci [26–28]. Polyadenylation is a co-transcriptional process whereby recognition of core motifs by the cleavage and polyadenylation (CPA) complex triggers RNA cleavage, deposition of a poly(A) tail on the 3′ end of the pre-mRNA, and transcription termination through XRN-mediated 5′→3′ degradation of leftover nascent RNA from RNA polymerase II [29–32]. A single gene can contain multiple core polyadenylation motifs, enabling alternative polyadenylation (APA) site usage that manifests either as canonical 3′UTR polyadenylation or ‘early-terminated’ intronic polyadenylation.

Previous studies have shown that defects in the polyadenylation machinery lead to transcription termination failure, causing RNA polymerase II to read through intended endpoints [18, 31, 33]. Additionally, genome-wide analyses have revealed that intragenic CpG island transcription is correlated with early termination of host genes [27]. However, the mechanistic demonstration of transcriptional interference at endogenous mammalian loci remains unclear as to whether transcription itself is a direct driver of termination, whether this results in intronic polyadenylation, and whether transcriptional read-through during polyadenylation failure affects transcriptional interference. Furthermore, given research indicating that DNA methylation influences polyadenylation and vice versa [34, 35], transcription and DNA methylation require uncoupling to demonstrate the impact, or lack thereof, of transcriptional interference on gene termination.

**Figure 1.**
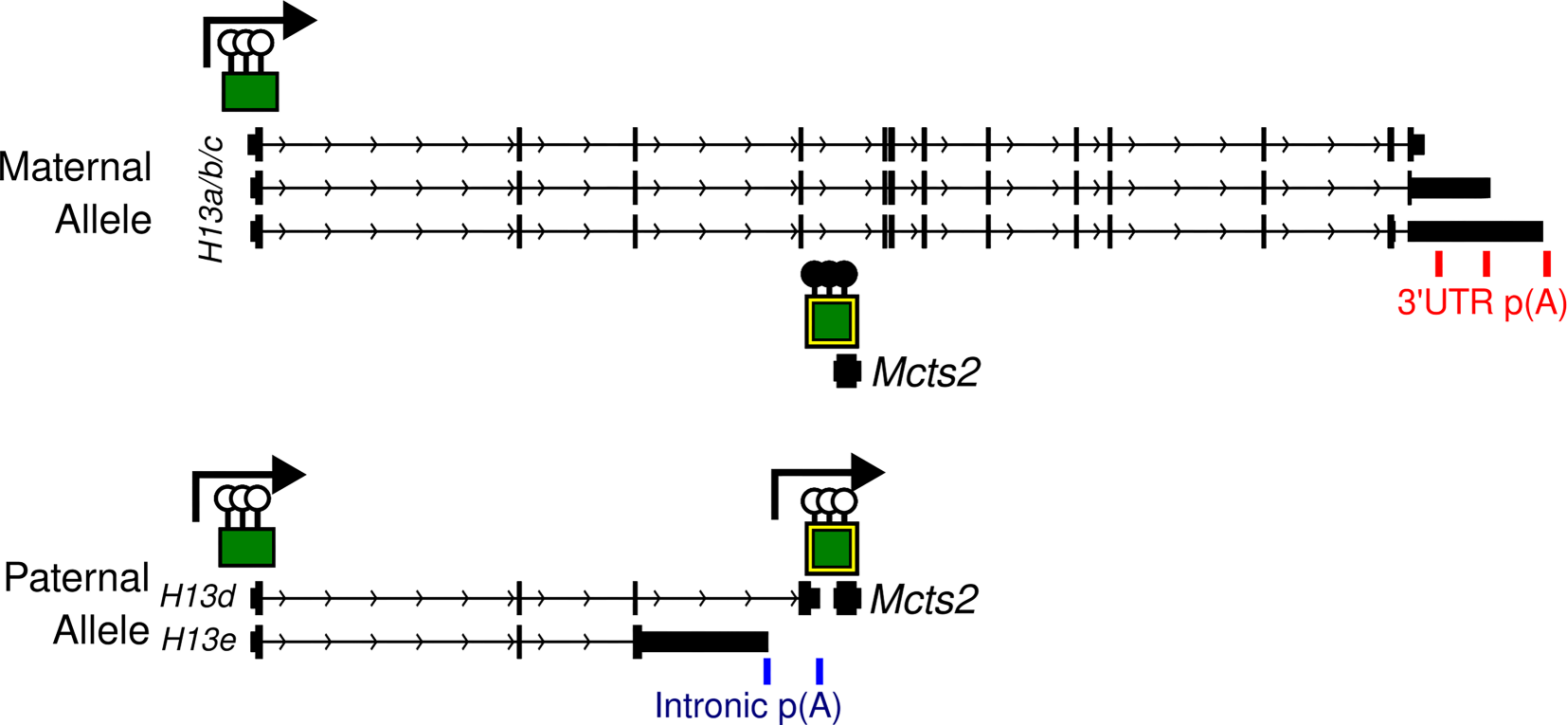
Schematic of the imprinted *H13/Mcts2* locus. Black lines show the transcripts at the locus, and each black rectangle is an exon of *H13* or *Mcts2*. The *Mcts2* CGI serves as the ICR (green square, yellow outline), filled black circles represent DNA methylation at this ICR and a lack of *Mcts2* expression, which is correlated with canonical 3′UTR polyadenylation site usage of *H13*, and is the case on the maternal allele. Conversely, the absence of DNA methylation at the ICR on the paternal allele, indicated by white circles, results in *Mcts2* expression and intronic polyadenylation of *H13. H13* Canonical 3′UTR polyadenylation sites are indicated by the red lines, and intronic polyadenylation site usage by the blue lines.

To investigate this relationship, we leverage the imprinted *H13/Mcts2* locus (illustrated in Fig. 1) to mechanistically probe the impact of both transcription and polyadenylation at this model host/nested gene pair. At this locus, Histocompatibility Minor 13 (*H13*) serves as the host gene for Malignant T Cell Amplified Sequence 2 (*Mcts2*), which originally retrotransposed from its ancestral gene, *Mcts1*, into the fourth intron of *H13* [36, 37]. The CGI promoter for *Mcts2* is imprinted and exhibits allele-specific DNA methylation and expression [36].

*H13* harbours five polyadenylation sites: two are intronic, located at introns 3 and 4, and the other three reside in its canonical 3′UTR. Maternal DNA methylation at the *Mcts2* CGI promoter and lack of *Mcts2* expression correlate with *H13*, exclusively utilising 3′UTR polyadenylation sites, transcribing the isoforms *H13a, H13b*, and *H13c*. However, on the paternal allele, where the *Mcts2* CGI promoter lacks DNA methylation, and *Mcts2* is expressed, *H13* utilises intronic polyadenylation sites, transcribing the isoforms *H13d* and *H13e* (Fig. 1) [38]. Thus, the consequence of an active and inactive nested gene can be examined in the exact same cellular context.

Using the *H13/Mcts2* locus as a model host/nested gene pair in hybrid C57BL/6 (B6) and CAST/EiJ (CAST) mouse neural stem cells (NSCs), we provide a mechanistic demonstration that *bona fide* transcriptional interference operates at an endogenous mammalian locus, driving intronic polyadenylation independently of DNA methylation. Furthermore, even without a functional polyadenylation site, host gene transcription is unable to continue through *Mcts2*, suggesting that transcriptional interference can terminate host gene transcription with or without the option of polyadenylation.

## Methods

### Neural Stem Cell Culture

Hybrid C57BL/6 / CAST/EiJ mouse NSCs were extracted from the subventricular zone and expanded as previously described [39]. NSCs were cultured in N2B27 media + Fgf2 + Egf: DMEM/F12 [Gibco, 11 320 033]: Neurobasal [Gibco, 21 103 049] at a 1:1 ratio, N2 1X final [Millipore, SCM012], B27 0.5x final [Gibco, 17504- 044], L-Glutamine 1x final [Gibco, 25030–024], and 2-mercaptoethanol 0.1mM [Gibco, 31350–010]. FGF2 [Peprotech, 100–18B] and EGF [Peprotech, 315–09] were reconstituted in PBS + 0.1% BSA, and 1 µl of each growth factor was added per 10 ml of N2B27 media to a final concentration of 10ng/ml. Cell culture plates were coated with 1/100^th^ diluted Geltrex^TM^ LDEV-Free Reduced Growth Factor Basement Membrane Matrix [Gibco, A1413201] in DMEM-F12 media prior to NSC plating. Cells were detached using Accutase [Millipore, SCR005] and passaged every 2–3 days when reaching 80% confluency. Cells being recovered from liquid nitrogen storage were cultured for one day in N2B27 + Fgf2 + Egf + RevitaCellTM Supplement 1X [ThermoFisher, A2644501]. Clonal isolation was performed by plating NSCs at a very low density of 3000 cells per 10 cm dish. Individual colonies were manually picked using a P20 pipette and transferred to a 96-well cell culture plate.

### DNA & RNA extraction

DNA was extracted from NSCs using Phenol/Chloroform extraction. NSC pellets of 500 000 cells were resuspended in 500 µl lysis buffer: 100 mM Tris pH 8.0, 5 mM EDTA, 0.2% SDS, 50 mM NaCl, 0.05 mg/ml Proteinase K, and 0.05mg/ml RNase A. The resuspended cell pellet was incubated at 50°C for 30 min. Equal volume of UltraPure^TM^ Phenol:Chloroform:Isoamyl Alcohol [Invitrogen, 15 593 031] was added, mixed, and transferred to a phase lock tube [QuantaBio, 2 302 830] for aqueous phase separation following the manufacturer’s instructions. DNA was precipitated using isopropanol precipitation and resuspended in nuclease-free water. For clonal DNA extraction in 96-well plates, 100 µl of lysis buffer was added and incubated overnight at 37°C. 100 µl of 100% isopropanol was added for 1 h and incubated at room temperature to precipitate the DNA. The precipitated DNA was pelleted, washed with 70% Ethanol, and dried for 1 h. 80 µl of nuclease-free water was added to each well, and the DNA pellet was dissolved overnight at 37°C. Around 1 µl of this solution was used per PCR for colony genotyping. RNA was extracted using 1 ml TRIzolTM Reagent [ThermoFisher, 15 596 026] added directly to NSCs cell culture plates. Purified RNA was DNase-treated using TURBO DNA-freeTM Kit [Invitrogen, AM1907].

### dCas9:KRAB cell line generation

LentiX cells were plated onto a 10 cm cell culture dish and grown to 80% confluency. Equimolar ratios of psPAX2, pMD2.G, and the donor plasmids were transfected into LentiX cells using jetOPTIMUS® DNA Transfection Reagent (Polyplus: 101 000 051) as per the manufacturer’s instructions. Donor plasmids used were: pLKO5.sgRNA.EFS.GFP (Addgene: #57 822) and a dCas9:KRAB:mCherry plasmid [40] (provided by Claire Rougeulle). Supernatant containing lentiviruses was collected at 48- and 72-h post-transfection and filtered through a 45 µM filter. Lentiviruses were then concentrated overnight at 3000 x G at 4°C, and the viral pellet was dried. The viral pellet was resuspended in 100 µl of DMEM-F12, and 10 µl of this solution was added to the growth media and added directly to NSCs. This was removed 24 h later, and transduced NSCs were detached and validated by sorting using flow cytometry.

### CRISPR knockout cell line generation

To generate a poly(A) deletion, two gRNA were designed targeting an 84 bp region containing the *H13d* polyadenylation motifs: UGUA, AATAAA, and a T/U-Rich region. gRNAs (crRNA) were cloned into a pLKO5.sgRNA.EFS.GFP plasmid via restriction ligation cloning. PCR products that amplified the gRNA (crRNA + tracrRNA) with a 5′ T7 promoter overhang were used as templates for *in-vitro* transcription to generate the gRNAs. 10µg Alt-R S.p. Cas9 Nuclease V3 [IDT] was incubated with the gRNA at room temperature for 10 min. NSCs were prepared for electroporation using the Mouse Neural Stem Cell Nucleofector® Kit [Lonza, VPG- 1004]. Electroporation was performed using the Amaxa Nucleofactor II, T-030 program. NSCs were plated with N2B27 + EGF & FGF2 + RevitaCellTM Supplement 1X [ThermoFisher, A2644501] for 1 day as a recovery medium, which was refreshed after 4 h with N2B27 + EGF & FGF2.

### PCR, cDNA synthesis, qPCR & Sanger sequencing

Primers were designed using Primer 3 in Benchling. PCR was performed using 20ng of genomic DNA per reaction. PCR was performed using VeriFi Red mix [PCR Biosystems, PB10.44–01]. cDNA synthesis was performed using the UltraScript® Reverse Transcriptase & cDNA Synthesis Kit [PCR Biosystems, PB30.11–02], which is a combination of random hexamers and oligo dT. cDNA synthesis was performed for polyadenylation knock-outs using oligo dT or random hexamers using Maxima H Minus Reverse Transcriptase [ThermoScientific, EP0751]. qPCR was performed in a 384-well plate (QuantStudio6) with a total reaction volume of 5µl using the qPCRBIO SyGreen Mix Lo-ROX [PCR Biosystems, PB20.11–50]. Relative expression was calculated using three housekeeping genes: *Ppia, Rpl30, Tbp*, and using the following equation: 2^-AverageCt Housekeeping genes^ / 2^-AverageCt Test gene^. Fold change was calculated using the ddCT method. PCR and qPCR amplicons intended for Sanger Sequencing were purified using the DNA Clean & Concentrator-5 [Zymo, D4014]. GeneWiz/Azenta Life Sciences provided the Sanger Sequencing service. Sanger sequencing was visualised using QIAGEN CLC genomics workbench. Primers used for these assays are detailed in Supplemental Table 1.

### DNA methylation assessment

EZ DNA Methylation-Gold Kit [Zymo, D5006] was used to bisulfite convert genomic DNA. Converted DNA was then utilised to perform PCR using HotStarTaq DNA Polymerase [Qiagen, 203 203] and the PCR product cleaned using the DNA Clean & Concentrator-25 kit [Zymo, D4033]. PCR products were A-tailed and cloned into a pGEM-T easy vector [Promega, A1360] for Sanger sequencing. Primers used for these assays are detailed in Supplemental Table 1.

### 3′RACE and 3′RACE sequencing analysis

3′RACE was performed using the SMARTer® RACE 5′/3′ Kit [Takara, 634 858] using the manufacturer’s instructions. The PCR step was performed using Q5® High-Fidelity DNA Polymerase [M0491L]. PCR cycling conditions were as follows for each 3′RACE. Exon4 3′RACE: 1 min 98°C, 98°C 10 s, 66°C 20, 72°C 1 min for 25 cycles with 72°C 10 min final extension. Exon2 3′RACE was performed as above with 60°C annealing temperature and a follow-up nested PCR using a 66°C annealing temperature with 17 PCR cycles. Premium PCR Sequencing was performed by Plasmidsaurus using Oxford Nanopore Technology. Reads were manually counted at the maternal (B6) or paternal (CAST) allele if a single long read spanned two known SNPs. Long reads were aligned using minimap2 [41] and converted to bam files using samtools [42]. Primers used for these assays are detailed in Supplemental Table 1.

### CUT&RUN

CUT&RUN was performed on freshly detached NSCs following the protocol detailed in [43] with some modifications detailed here. Activation buffer and Concanavalin A beads were purchased from Cell Signalling Technologies [#93 569]. Digitonin concentration utilised was at 5% across all buffers, and MNase digestion time was at 30 min on ice. Recovery of DNA fragments using each method was confirmed using Qubit dsDNA HS Assay [Invitrogen, Q32851]. Around 10 ng of this DNA was utilised for the generation of CUT&RUN libraries using the protocol in (Liu, 2019). Libraries were generated using NEBNext® UltraTM II DNA Library Prep Kit for Illumina® [E7645S] and indexed using NEBNext® Mtiplex Oligos for Illumina® (Index Primers Set 1) [E7335S]. 1µl of 3µM of NEBNext Adaptor was utilised to reduce adaptor dimers. Sera-Mag select beads [Cytiva, 29 343 052] were used for DNA clean-up and size selection. DNA Library size was measured using Agilent High Sensitivity D1000 ScreenTape [Agilent, 5067- 5585]. Antibodies utilized were: H3K4me3 [Abcam, ab8580], H3K36me3 [EpiCypher, 13–0058].

### Bioinformatic analysis

Adapters were trimmed from fastq reads using trim-galore. Fastq files were aligned using bowtie2 [44] to a N-Masked mouse genome (mm39). The N-Masked genome file was generated using SNPSplit (Krueger and Andrews, 2016), which required B6/CAST SNP information (.vcf) and the mm39 fasta genome sequence file. Once aligned, the aligned reads were then split into B6 (maternal) and CAST (paternal) read files for processing into .bigWigs using deeptools [45]. Reads upstream and downstream of *Mcts2* were counted using featureCounts with a custom .saf file from the aligned and split .bam file. RPKM normalisation was performed on aligned reads mapping to chr2 to alleviate normalisation artefacts derived from other chromosomes.

## Results

### Transcription is the main driver of intronic *H13* polyadenylation

To assess the impact of nested transcription as the driver of intronic polyadenylation at the *H13/Mcts2* locus, we leveraged male NSCs derived from hybrid evolutionarily distinct strains of mice (C57BL/6 (B6) and CAST/EiJ (CAST)). The *H13/Mcts2* locus harbours multiple SNPs between these strains throughout the region, providing a means to determine the parental origin of DNA methylation at the imprinting control region (ICR), and allelic transcript and histone modifications. Each SNP can be allocated to either the maternal or paternal allele, enabling precise tracking of allele-specific effects (llustrated in Supplemental Figure 1A). Our NSC model also contains the appropriate imprinting throughout multiple passages in cell culture, demonstrating allelic DNA methylation at the ICR, maternal *H13abc* and paternal *H13d, H13e* and *Mcts2* transcripts at both early and late passages (Supplemental Fig. 1B–C). Furthermore, this cell line supports allele-specific CUT&RUN to map H3K4me3 (promoter-associated) and H3K36me3 (elongation-associated) histone modifications at high resolution against either the maternal or paternal genome (Supplemental Fig. 2A–C).

**Figure 2.**
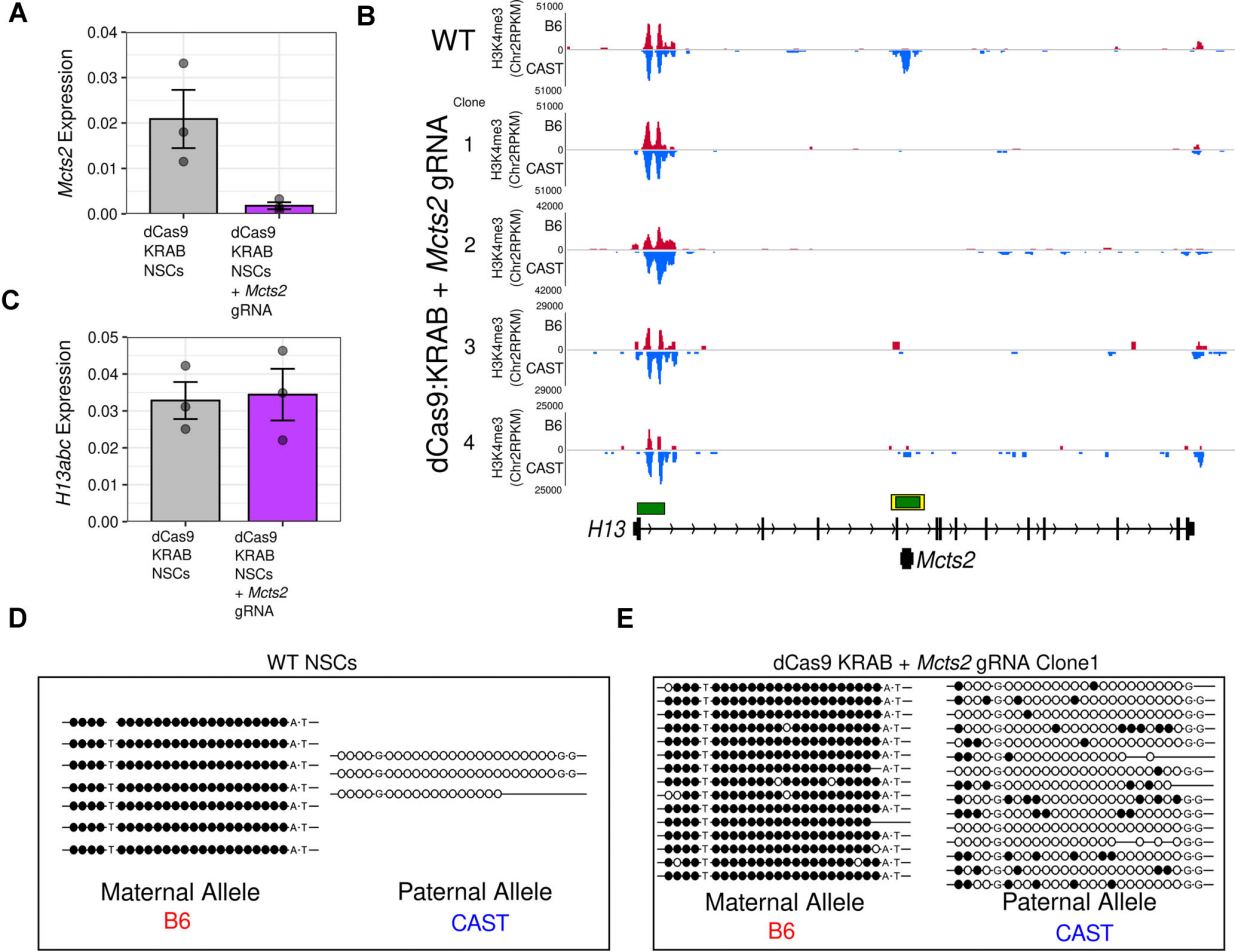
Active transcription of *Mcts2* drives intronic polyadenylation of host gene *H13* through transcriptional interference. (**A**) Confirmation of successful dCas9:KRAB-mediated suppression of *Mcts2* via qPCR, *n* = 3 Student’s t-test *P* < 0.05. (**B**) Allelic H3K4me3 CUT&RUN of NSC clones with silenced *Mcts2* allele demonstrated loss of paternal H3K4me3 at the *Mcts2* promoter. Tracks represent RPKM normalised to Chromosome 2. (**C**) dCas9:KRAB-mediated suppression of *Mcts2* did not change *H13abc* expression levels. (**D** and **E)** dCas9:KRAB suppression did not lead to widespread DNA methylation at the *Mcts2* promoter, demonstrated via bisulfite PCR, cloning, and Sanger sequencing analysis at the *Mcts2* promoter/ICR. Circles represent individual CpGs, black = methylated, white = unmethylated.

**Figure 3.**
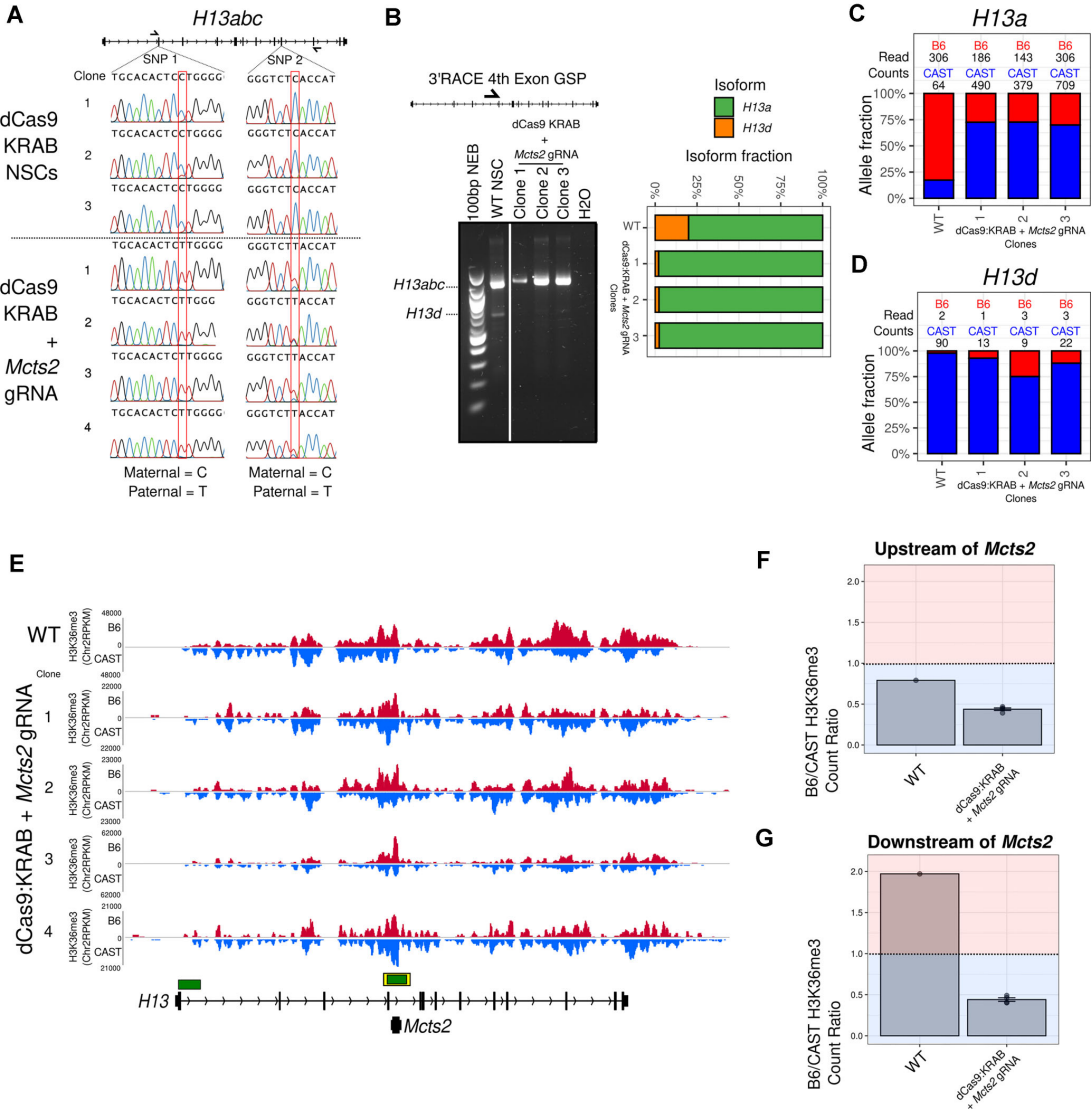
Active transcription of *Mcts2* drives intronic polyadenylation of host gene *H13* through transcriptional interference. (**A**) *Mcts2* suppression resulted in paternalisation of *H13abc* transcripts, illustrated by Sanger sequencing traces of an RT-PCR product containing two SNPs, both whereby C = Maternal and T = Paternal. (**B**) 3′RACE using a forward primer from *H13* Exon 4 shows that *Mcts2* repression reduces intronic polyadenylation of *H13d, as* shown by agarose gel (left) and long-read amplicon sequencing of 3′RACE products (right). (**C**) Allelic analysis of 3′RACE amplicon products demonstrates that Mcts2 silencing shifts H13abc to paternal (blue) than maternal (red) expression. (**D**) *H13d* remains mainly paternal (blue) when *Mcts2* is silenced, and small maternal expression indicates a rare event of dCas9:KRAB inducing intronic polyadenylation at the *H13/Mcts2*. (**E**) Allelic CUT&RUN demonstrated that elongation marker, H3K36me3, extends beyond the *Mcts2* promoter at *H13/Mcts2* in the *Mcts2*-suppressed NSC lines. Barplots of H3K36me3 tracks show ratios of B6/CAST H3K36me3 reads (**F**) upstream and (**G**) downstream of *Mcts2*. Thus, a value > 1 indicates more maternal H3K36me3 signal than paternal in the illustrated windows, and vice versa for values < 1.

To investigate the impact of nested transcription on host elongation, we used a dCas9-KRAB system for targeted gene repression, whereby loading of a gRNA to the dCas9 can target KRAB-mediated H3K9me3 deposition to a desired promoter. A stable NSC line was generated with a gRNA targeting the *Mcts2* promoter to specifically repress *Mcts2* expression. Across three independent clones, expression of dCas9:KRAB and an *Mcts2* targeting gRNA in NSCs resulted in a significant decrease in *Mcts2* expression (Fig. 2A). Chromatin profiling using allelic CUT&RUN confirmed that the decrease in expression of *Mcts2* was associated with a withdrawal of H3K4me3 from the paternal *Mcts2* promoter (Fig. 2B). *Mcts2* repression did not result in a change in total *H13abc* transcript levels (Fig. 2C), and DNA methylation remained largely absent from the paternal *Mcts2* promoter, similar to WT NSCs (Fig. 2D and E). Whilst total transcript levels of *H13abc* remained consistent despite *Mcts2* suppression, allele-specific analysis of RT-PCR products revealed enhanced *H13abc* expression from the paternal allele vs the maternal allele (Fig. 3A). This was confirmed by 3′RACE experiments, where we observed a higher proportion of *H13abc* and a lower proportion of *H13d* when *Mcts2* was repressed (Fig. 3B), showing that less intronic polyadenylation was now occurring. Allelic mapping of the 3′RACE products also confirmed that the majority of *H13abc* were now expressed from the paternal allele, whereas the few *H13d* transcripts detected were still expressed from the paternal allele (Fig. 3C and D, Supplemental Fig. 3). 3′RACE performed using a primer initiating from Exon2 of *H13* and capturing intronic polyadenylation of *H13e* showed that *H13e* was also reduced, similar to *H13d*, when *Mcts2* was repressed (Supplemental Fig. 4A–D). Using H3K36me3 as a proxy for elongation, the ratio of H3K36me3 CUT&RUN reads mapping upstream and downstream of *Mcts2* to the maternal and paternal allele was calculated (Fig. 3E), revealing two key findings. First, upstream of *Mcts2*, paternal elongation was more frequent in both WT NSCs and in NSCs with a silenced *Mcts2* gene (Fig. 3F). Second, downstream of *Mcts2*, where elongation is mainly maternal in WT NSCs, there was a shift to more paternal elongation in NSCs with a silenced *Mcts2* gene (Fig. 3G). Thus, we demonstrate that nested gene transcription alone and *in cis* influences the elongation of another distinct host gene transcriptional event, indicative of elongation transcriptional interference independent of DNA methylation.

### Polyadenylation is not required for *H13* termination

Given that *Mcts2* transcription promotes *H13d* intronic polyadenylation site usage, we next sought to investigate whether prevention of polyadenylation via deletion of the recognition sequence would impact nested *Mcts2* transcription and whether *H13* transcription termination could still occur without polyadenylation. Prevention of polyadenylation in various cell line models leads to transcriptional read-through, extending transcription beyond the intended polyadenylation site use [18, 31, 46]. Under this hypothesis, transcriptional read-through would extend *H13* elongation through the *Mcts2* promoter, depositing H3K36me3 and subsequently recruiting DNMT3B-mediated DNA methylation to silence *Mcts2* [21, 22]. To investigate this, we used clonal NSC lines with paternal-specific deletions of the *H13d* polyadenylation site motifs (UGUA + AATAAA + U-rich region). We established two independent clones with paternal deletion, referred to as *ΔH13d poly(A)^±^* Clone A3 and Clone F6 (Supplementary Fig. 5A and B).

**Figure 4.**
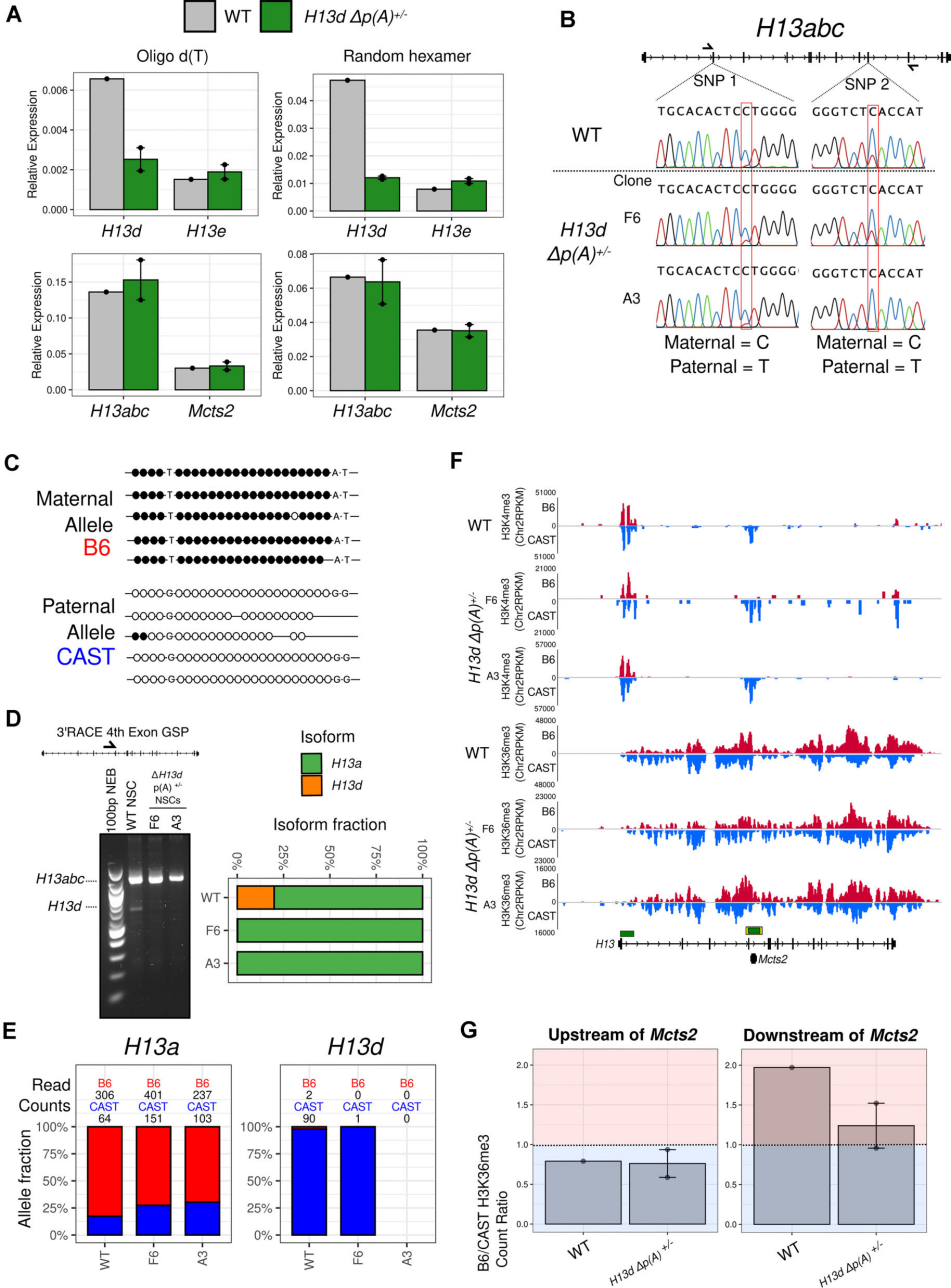
Nested *Mcts2* transcription is unaffected by reduction in host gene intronic polyadenylation. (**A**) Confirmation that genetic deletion of the *H13d* poly(A) sites results in a loss of *H13d* transcript level in both oligo d(T) and random hexamer converted RNA, with no change in *H13abc, Mcts2*, or *H13e* transcript levels. (**B**) Canonical 3′UTR transcript, *H13abc*, continued to be maternally expressed as shown by Sanger sequencing traces of RT-PCR products, which contain two B*C SNPs (C = maternal, T = paternal). (**C**) Further to this, DNA methylation analysis in *ΔH13d poly (A)^±^* NSC Clone F6 via bisulfite PCR, cloning, and Sanger sequencing analysis revealed no change in DNA methylation. Circles are individual CpGs, black = methylation, white = unmethylated. (**D**) *H13d* is absent from polyadenylated isoforms determined by 3′RACE in both *H13d* poly(A) knockout clones. (**E**) Knockout of *H13d* has minimal impact on the allelic polyadenylation of *H13abc* and remains maternally expressed. (**F**) Deletion of the *H13d* poly(A) site did not alter H3K4me3 at *Mcts2*, or impact deposition of the elongation marker H3K36me3 across the *H13/Mcts2* locus. (**G**) Ratio of H3K36me3 CUT&RUN reads upstream and downstream of Mcts2 was unaffected by *H13d* poly(A) site deletion.

In both *ΔH13d poly(A)^±^* clones, there was a 75% decrease in *H13d* expression, while expression of the other transcripts (*H13abc, H13e*, or *Mcts2*) remained unchanged (Fig. 4A). These findings were consistent across both random hexamer and oligo(dT) converted cDNA synthesis protocols (Fig. 4A). Importantly, allele-specific expression patterns were mainly unchanged compared to wild-type cells. *H13abc* continued to be predominantly expressed from the maternal allele, and *Mcts2* expression remained specific to the paternal allele in the *ΔH13d poly(A)^±^* clones (Fig. 4B), matching the WT state of the locus in NSCs. DNA methylation patterns were completely unaffected in the *ΔH13d poly(A)^±^* F6 clone at the ICR (Fig. 4C). 3′RACE experiments confirmed complete prevention of *H13d* intronic polyadenylation (Fig. 4D, Supplemental Fig. 6A) without having any impact on *H13e* expression (Supplemental Fig. 6B–D). *H13abc* expression was predominantly maternal when the *H13d* poly(A) site was deleted (Fig. 4E). Chromatin profiling in the *ΔH13d poly(A)^±^* clones demonstrated that elongation-associated H3K36me3 was still predominantly maternal downstream of *Mcts2*, remained unchanged upstream of *Mcts2*, and promoter-associated H3K4me3 remained at the intragenic *Mcts2* promoter (Fig. 4F and G). This suggests that even without a functional polyadenylation site, *Mcts2* transcription itself is sufficient to terminate host gene elongation, given that the locus maintained expected allelic expression and elongation in the *ΔH13d poly(A)^±^* clones, and that *Mcts2* expression remained unchanged with its CpG island promoter devoid of DNA methylation.

Collectively, our results demonstrate that *Mcts2* transcription can effectively terminate incoming host gene transcription even in the absence of a functional polyadenylation site, indicating that transcriptional interference itself, rather than polyadenylation machinery, drives transcription termination at this locus. We found no evidence of transcriptional read-through occurring through *Mcts2* on the paternal allele in the *ΔH13d poly(A)^±^* NSCs. These findings reveal that nested gene transcription can serve as an effective transcriptional barrier to host elongation, independent of polyadenylation-mediated termination.

## Discussion

**Figure 5.**
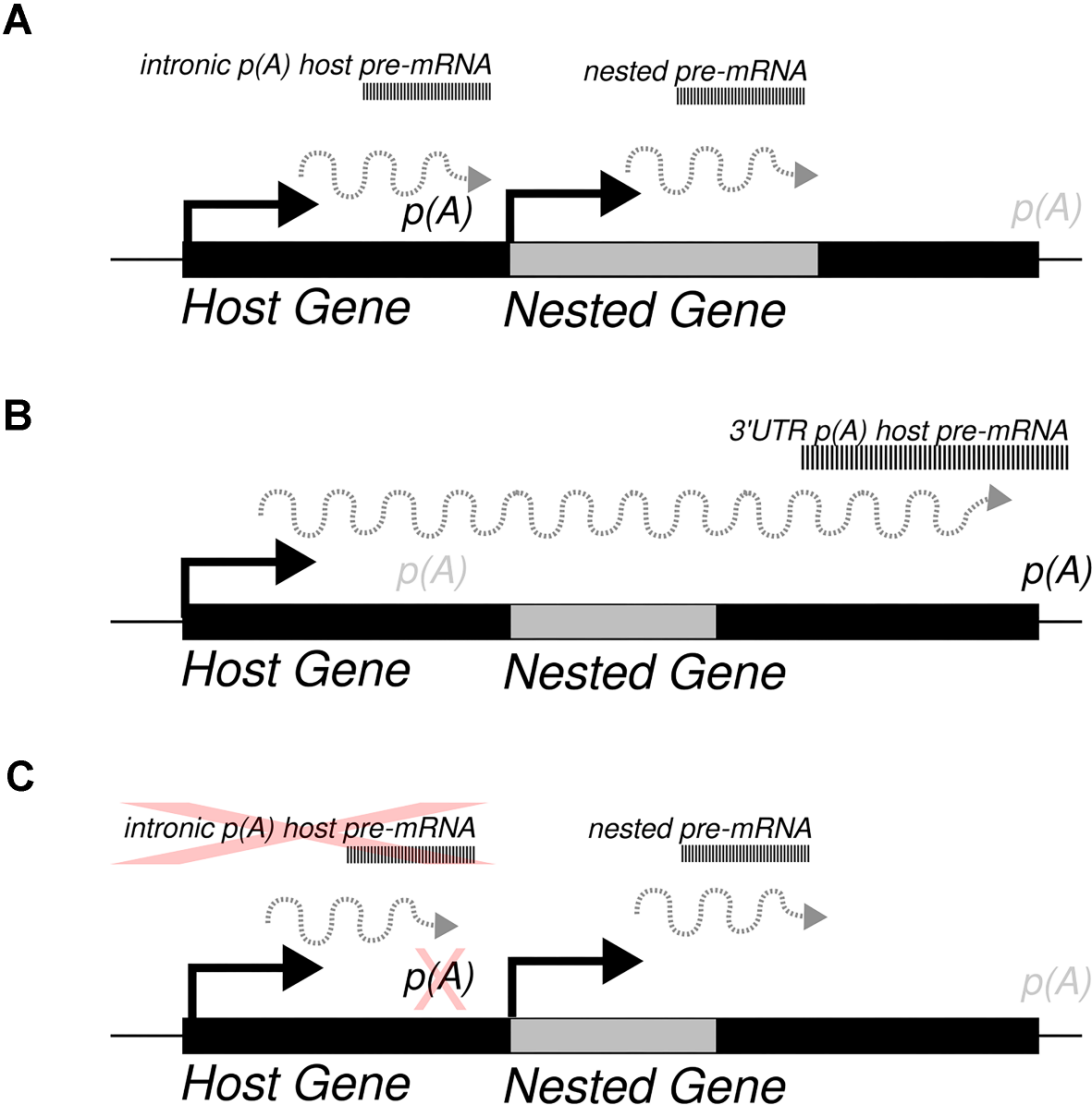
Outcomes of transcriptional interference at host/nested gene pairs. (**A**) Intronic polyadenylation of the host gene (black rectangle) as a polyadenylation site is proximal to the nested gene (grey rectangle) transcription. (**B**) Silenced nested gene expression removes the transcriptional roadblock, and host gene elongation continues to the canonical 3′UTR polyadenylation site. (**C**) When no polyadenylation site is available for the host gene, nested transcription instead causes non-productive host gene transcription, and the resultant host pre-mRNA is not stably expressed and is effectively downregulated.

Approximately 10% of all genes in mammals exhibit a host/nested gene spatial organisation whereby one gene exhibits complete genic overlap with another [19]. The consequences of such an arrangement, which creates potential for two distinct transcriptional events to collide, remain unclear. Within this work, the *H13/Mcts2* locus was used as a model to mechanistically demonstrate transcriptional interference at an endogenous mammalian locus and understand its consequences at host/nested gene pairs. By suppressing *Mcts2* without changing DNA methylation at the CGI promoter, we demonstrated that transcription itself is the main driver of intronic polyadenylation at *H13* on the paternal allele, because suppressing *Mcts2* results in paternalisation of the canonical *H13abc* transcript terminating at the 3′UTR polyadenylation sites (Figs 2 and 3). Thus we provide a mechanistic follow-up to our previous article, whereby we demonstrate that genome-wide, intragenic CpG island activity is highly correlated to increased termination [27]. Furthermore, deletion of the predominantly used intronic polyadenylation site that generates the paternal *H13d* transcript resulted in no changes to the expression of either the canonical *H13abc*, the nested *Mcts2*, or the upstream *H13e* transcripts (Fig. 4). These findings imply that transcriptional interference from *Mcts2* can terminate host gene transcription with or without the presence of an intronic polyadenylation. Thus, at other host/nested gene pairs, transcriptional interference at host/nested gene pairs is likely to either cause intronic polyadenylation if a site is available or generate unstable pre-mRNA, effectively downregulating the host gene (see model in Fig. 5).

This is in agreement with genome-wide analyses that previously identified an increase in RNA-sequencing reads upstream of active intragenic CpG islands [27]. Thus, we hypothesise that across the genome, regions of ‘strong’ nested transcription serve as sites where host gene transcription is susceptible to transcriptional interference. Previous mechanistic work has illustrated that the relative ‘strength’ of gene promoters can influence the ability of *LUC7L* to repress promoters via transcription-mediated repression [20]. Therefore, *Mcts2* appears to function as a ‘strong’ gene promoter whereby any incoming transcription will be terminated either through intronic polyadenylation or, if polyadenylation sites are unavailable, through an alternative termination mechanism. Indeed, variability in expression of intragenic CpG islands that are linked to intronic polyadenylation events was independent of DNA methylation [27], suggesting that such ‘strength’ is mediated by other factors.

A key finding of this study is the absence of DNA methylation at the *Mcts2* ICR despite KRAB-mediated repression. We found this surprising, given that endogenous KRAB-ZNF proteins typically repress retroelements through H3K9me3 deposition followed by stable DNA methylation [47–50, 51]. At ICRs, H3K9me3 is typically present on already-methylated alleles, and KRAB-ZNFs are instead important for DNA methylation maintenance as opposed to *de novo* methylation [52–54, 55]. In contrast, in this study, H3K9me3 is artificially recruited to the unmethylated *Mcts2* ICR via dCas9:KRAB. Additionally, unmethylated ICRs are actively protected from methylation through persistent recruitment of TET demethylases, which convert nascent 5mC to 5hmC, preventing stable DNA methylation establishment [56, 57, 58]. Furthermore, dCas9:KRAB methods alone cannot induce DNA methylation-mediated silencing without co-recruitment of DNMT3A and DNMT3L [59, 60, 61]. Therefore, the sustained repression of *Mcts2* without DNA methylation reflects the ability of dCas9:KRAB to silence transcription via H3K9me3 deposition. However, stable DNA methylation is likely not established due to both the lack of DNMT3A/L co-recruitment and TET-mediated protection at the unmethylated *Mcts2* ICR allele. Such technical and biological constraints successfully enabled a method to independently assess nested transcription on host gene elongation, independent of intragenic promoter DNA methylation.

An intriguing result from this work was that *Mcts2* repression led to no total changes of *H13abc* expression and that despite an increase in paternal expression, a subsequent decrease in maternal *H13abc* balanced the total levels to be the same as in WT NSCs (Fig 2C and 3). We hypothesise that *H13abc* is tightly regulated by a gene or enhancer network outside the locus to maintain a consistent dosage of *H13abc* transcript levels. Further manipulation of the locus will be required to untangle the network of genes involved in the regulation of *H13/Mcts2*.

Previous research has also demonstrated that the respective orientations of RNA polymerase II collisions may impact dynamics at host/nested gene pairs. Studies leveraging synthetic constructs reveal that ‘head-on’ collisions are prone to termination [8], while same-orientation collisions typically push RNA polymerase II along a gene rather than terminating transcription [9]. At the *H13/Mcts2* locus, the transcribing polymerases are both in the sense orientation, yet transcriptional interference still occurs, resulting in intronic polyadenylation of *H13d* on the paternal allele. While this may appear to contradict the concept that same-orientation collisions do not terminate transcription, it is worth noting that in mice harbouring a Chromosome 2 uniparental paternal duplication, small traces of the canonical *H13abc* transcript are observed despite *Mcts2* being active on both maternal and paternal alleles [38]. Therefore, the transcriptional interference at *H13/Mcts2* is likely not one hundred percent efficient, though it remains unclear whether this partial efficiency stems from the sense-sense orientation of RNA polymerase II collision, or from the dynamics of RNA Polymerase II loading and release at the *Mcts2* promoter in real-time [62, 63]). Related work has shown that recruitment of dCas9 in the antisense orientation, as opposed to the sense orientation, results in transcription elongation defects [64]. The gRNA utilised for dCas9 recruitment to the *Mcts2* promoter (Figs 2 and 3) is in the sense orientation, which might explain why, despite the recruitment of KRAB-mediated repression machinery, RNA polymerase II was still capable of elongating. In agreement with this observation, only minimal termination was induced due to dCas9:KRAB recruitment (Fig. 3D), where < 1% (an average of 3 read counts) of total *H13* transcript levels demonstrated this phenomenon. Independent of orientation, the precise stage of transcription that causes the observed interference remains unclear. Interference could occur during pre-initiation complex assembly, which spans ∼100 bp at CpG island promoters and results in steric hindrance with the incoming polymerase [65, 66]. Alternatively, bidirectional transcription, which is widespread at gene promoters [67, 68], could generate ‘head-on’ collisions that induce termination at host/nested genes.

The event of transcriptional interference not only has implications for host/nested gene pairs but also for the transcriptionally active intragenic transposable elements. Disruption to transcription has been observed due to TE insertions [26, 69], and may be far more widespread considering that 85–90% of introns contain TE sequences. Similar to *Mcts2*, which originated from retrotransposition of *Mcts1*, many TEs are embedded within introns of host genes and retain their own promoters, representing natural examples of nested transcriptional units that could function mechanistically similarly to the *H13/Mcts2* locus in regulating host gene expression through transcriptional interference. While the majority of TE sequences are either mutated or held transcriptionally silent via epigenetic silencing [70, 71], approximately 2% evade silencing and are transcriptionally active in non-coding regions, including introns and intergenic regions [72]. Notably, TEs are frequently activated during viral infection and early development, suggesting that the transcriptional interference mechanism we observed at *H13/Mcts2* may be widely relevant during these critical biological processes [73, 74].

## Supplementary Material

gkag640_Supplemental_File

## Data Availability

CUT&RUN data are deposited at GEO: GSE308559.

## Notes

Current address: Université Clermont Auvergne, CNRS, Inserm, iGReD, Clermont-Ferrand 63001, France

## References

[1] Haberle V, Stark A. Eukaryotic core promoters and the functional basis of transcription initiation. Nat Rev Mol Cell Biol. 2018;19:621–37. 10.1038/s41580-018-0028-8. 29946135 PMC6205604

[2] Mugridge JS, Coller J, Gross JD. Structural and molecular mechanisms for the control of eukaryotic 5′–3′ mRNA decay. Nat Struct Mol Biol. 2018;25:1077–85. 10.1038/s41594-018-0164-z. 30518847

[3] Mitschka S, Mayr C. Context-specific regulation and function of mRNA alternative polyadenylation. Nat Rev Mol Cell Biol. 2022;23:779–96. 10.1038/s41580-022-00507-5. 35798852 PMC9261900

[4] Shearwin KE, Callen BP, Egan JB. Transcriptional Interference - A Crash Course. Trends Genet. 2005; 21:339–45. 10.1016/j.tig.2005.04.009. 15922833 PMC2941638

[5] Wright BW, Molloy MP, Jaschke PR. Overlapping genes in natural and engineered genomes. Nat Rev Genet. 2022;23:154–68. 10.1038/s41576-021-00417-w. 34611352 PMC8490965

[6] Hobson DJ, Wei W, Steinmetz LM et al. RNA polymerase II collision interrupts convergent transcription. Mol Cell. 2012;48:365–74. 10.1016/j.molcel.2012.08.027. 23041286 PMC3504299

[7] Soulavie F, Couillault C, Melki M et al. Induction of host genes by nested genes during C. elegans development. iScience. 2025;28:113021, 10.1016/j.isci.2025.113021. 40686608 PMC12274878

[8] Wang L, Watters JW, Ju X et al. Head-on and co-directional RNA polymerase collisions orchestrate bidirectional transcription termination. Mol Cell. 2023;83:1153–64. 10.1016/j.molcel.2023.02.017. 36917983 PMC10081963

[9] Saeki H, Svejstrup JQ. Stability, flexibility, and dynamic interactions of colliding RNA polymerase II elongation complexes. Mol Cell. 2009;35:191–205. 10.1016/j.molcel.2009.06.009. 19647516 PMC2791892

[10] Martens JA, Laprade L, Winston F. Intergenic transcription is required to repress the Saccharomyces cerevisiae SER3 gene. Nature. 2004;429:571–4. 10.1038/nature02538. 15175754

[11] Nevers A, Doyen A, Malabat C et al. Antisense transcriptional interference mediates condition-specific gene repression in budding yeast. Nucleic Acids Res. 2018;46:6009–25. 10.1093/nar/gky342. 29788449 PMC6158615

[12] Alvarez JJC, Revel M, Carrasco J et al. Repression of the Hox gene abd-A by ELAV-mediated transcriptional interference. PLoS Genet. 2021;17:e1009843. 10.1371/journal.pgen.1009843. 34780465 PMC8629391

[13] Corbin V, Maniatis T. Role of transcriptional interference in the Drosophila melanogaster Adh promoter switch. Nature. 1989;337:279–82. 10.1038/337279a0. 2492088

[14] Jorgensen V, Chen J, Vander Wende H et al. Tunable transcriptional interference at the endogenous alcohol dehydrogenase gene locus in drosophila melanogaster. G3 GenesGenomesGenetics. 2020;10:1575–83. 10.1534/g3.119.400937. PMC7202008 32213532

[15] Rosa S, Duncan S, Dean C. Mutually exclusive sense–antisense transcription at FLC facilitates environmentally induced gene repression. Nat Commun. 2016;7:13031. 10.1038/ncomms13031. 27713408 PMC5059766

[16] Glotzer GL, Pastor PDH, Kronauer DJC. Transcriptional interference gates monogenic odorant receptor expression in ants. Curr Biol. 2025;35:5033–47. 10.1016/j.cub.2025.09.026. 40975052 PMC12493986

[17] Latos PA, Pauler FM, Koerner MV et al. Airn transcriptional overlap, but not its lncRNA products, induces imprinted Igf2r silencing. Science. 2012;338:1469–72. 10.1126/science.1228110. 23239737

[18] Prescott EM, Proudfoot NJ. Transcriptional collision between convergent genes in budding yeast. Proc Natl Acad Sci USA. 2002;99:8796–801. 10.1073/pnas.132270899. 12077310 PMC124378

[19] Montibus B, Cain JA, Martinez-Nunez RT et al. Global identification of mammalian host and nested gene pairs reveal tissue-specific transcriptional interplay. Genome Res. 2024;34:2163–75., , 10.1101/gr.279430.124. 39578100 PMC11694760

[20] Jeziorska DM, Murray RJS, De Gobbi M et al. DNA methylation of intragenic CpG islands depends on their transcriptional activity during differentiation and disease. Proc Natl Acad Sci USA. 2017;114:E7526–35. 10.1073/pnas.1703087114. 28827334 PMC5594649

[21] Neri F, Rapelli S, Krepelova A et al. Intragenic DNA methylation prevents spurious transcription initiation. Nature. 2017;543:72–7. 10.1038/nature21373. 28225755

[22] Teissandier A, Bourc’his D. Gene body DNA methylation conspires with H3K36me3 to preclude aberrant transcription. EMBO J. 2017;36:1471–3. 10.15252/embj.201796812. 28442531 PMC5452023

[23] Lin D, Hiron TK, O’Callaghan CA. Intragenic transcriptional interference regulates the human immune ligand MICA. EMBO J. 2018;37:EMBJ201797138. 10.15252/embj.201797138. PMC5978299 29643123

[24] Tufarelli C, Stanley JAS, Garrick D et al. Transcription of antisense RNA leading to gene silencing and methylation as a novel cause of human genetic disease. Nat Genet. 2003;34:157–65. 10.1038/ng1157. 12730694

[25] Williamson CM, Ball ST, Dawson C et al. Uncoupling antisense-mediated silencing and DNA methylation in the imprinted Gnas cluster. PLoS Genet. 2011;7:e1001347. 10.1371/journal.pgen.1001347. 21455290 PMC3063750

[26] Kaer K, Branovets J, Hallikma A et al. Intronic L1 retrotransposons and nested genes cause transcriptional interference by inducing intron retention, exonization and cryptic polyadenylation. PLoS One. 2011;6:e26099. 10.1371/journal.pone.0026099. 22022525 PMC3192792

[27] Amante S, Montibus B, Cowley M et al. Transcription of intragenic CpG islands influences spatiotemporal host gene pre-mRNA processing. Nucleic Acids Res. 2020;48:8349–59., 10.1093/nar/gkaa556. 32621610 PMC7470969

[28] Cain JA, Montibus B, Oakey RJ. Intragenic CpG islands and their impact on gene regulation. Front. Cell Dev. Biol. 2022;10:832348. 10.3389/fcell.2022.832348. 35223855 PMC8873577

[29] Proudfoot NJ. Ending the message: poly(A) signals then and now. Genes Dev. 2011;25:1770–82. 10.1101/gad.17268411. 21896654 PMC3175714

[30] Cortazar MA, Sheridan RM, Erickson B et al. Control of RNA Pol II speed by PNUTS-PP1 and Spt5 dephosphorylation facilitates termination by a “Sitting Duck Torpedo” mechanism. Mol Cell. 2019;76:896–908. 10.1016/j.molcel.2019.09.031. 31677974 PMC6927536

[31] Eaton JD, Francis L, Davidson L et al. A unified allosteric/torpedo mechanism for transcriptional termination on human protein-coding genes. Genes Dev. 2020;34:132–45. 10.1101/gad.332833.119. 31805520 PMC6938672

[32] Stroup EK, Ji Z. Deep learning of human polyadenylation sites at nucleotide resolution reveals molecular determinants of site usage and relevance in disease. Nat Commun. 2023;14:7378. 10.1038/s41467-023-43266-3. 37968271 PMC10651852

[33] Bauer DLV, Tellier M, Martínez-Alonso M et al. Influenza virus mounts a two-pronged attack on host RNA polymerase II transcription. Cell Rep. 2018;23:2119–29. 10.1016/j.celrep.2018.04.047. 29768209 PMC5972227

[34] Nanavaty V, Abrash EW, Hong C et al. DNA methylation regulates alternative polyadenylation via CTCF and the cohesin complex. Mol Cell. 2020;78:752–64. 10.1016/j.molcel.2020.03.024. 32333838 PMC7245569

[35] Fink EE, Nanavaty V, Lee BH et al. Heat shock induces alternative polyadenylation through dynamic DNA methylation-regulated chromatin looping. Cell Stress Chaperones. 2025;30:100084. 10.1016/j.cstres.2025.100084. 40412548 PMC12162027

[36] Wood AJ, Roberts RG, Monk D et al. A screen for retrotransposed imprinted genes reveals an association between x chromosome homology and maternal germ-line methylation. PLoS Genet. 2007;3:0192–203. 10.1371/journal.pgen.0030020. PMC1796624 17291163

[37] McCole RB, Loughran NB, Chahal M et al. A case-by-case evolutionary analysis of four imprinted retrogenes. Evolution. 2011;65:1413–27. 10.1111/j.1558-5646.2010.01213.x. 21166792 PMC3107425

[38] Wood AJ, Schulz R, Woodfine K et al. Regulation of alternative polyadenylation by genomic imprinting. Genes Dev. 2008;22:1141–6. 10.1101/gad.473408. 18451104 PMC2335310

[39] Ferrón SR, Andreu-Agulló C, Mira H et al. A combined *ex/in vivo* assay to detect effects of exogenously added factors in neural stem cells. Nat. Protoc. 2007;2:849–59. 17474182 10.1038/nprot.2007.104

[40] Furlan G, Hernandez NG, Huret C et al. The Ftx noncoding locus controls X chromosome inactivation independently of its RNA products. Mol Cell. 2018;70:462–72. 10.1016/j.molcel.2018.03.024. 29706539

[41] Li H. Minimap2: pairwise alignment for nucleotide sequences. Bioinformatics. 2018;34:3094–100. 10.1093/bioinformatics/bty191. 29750242 PMC6137996

[42] Li H, Handsaker B, Wysoker A et al. The sequence alignment/map format and SAMtools. Bioinformatics. 2009;25:2078–9. 10.1093/bioinformatics/btp352. 19505943 PMC2723002

[43] Janssens D, Henikoff S. CUT&RUN: targeted in situ genome-wide profiling with high efficiency for low cell numbers. Nat Protoc. 2018;13:1006–19. 10.1038/nprot.2018.015. 29651053

[44] Langmead B, Salzberg SL. Fast gapped-read alignment with Bowtie 2. Nat Methods. 2012;9:357–9. 10.1038/nmeth.1923. 22388286 PMC3322381

[45] Ramírez F, Dündar F, Diehl S et al. DeepTools: a flexible platform for exploring deep-sequencing data. Nucleic Acids Res. 2014;42:W187–91. 24799436 10.1093/nar/gku365 PMC4086134

[46] Orkin SH, Cheng TC, Antonarakis SE et al. Thalassemia due to a mutation in the cleavage-polyadenylation signal of the human beta-globin gene. EMBO J. 1985;4:453–6. 10.1002/j.1460-2075.1985.tb03650.x. 4018033 PMC554207

[47] Fukuda K, Makino Y, Kaneko S et al. Potential role of KRAB-ZFP binding and transcriptional states on DNA methylation of retroelements in human male germ cells. eLife. 2022;11:e76822. 10.7554/eLife.76822. 35315771 PMC8967385

[48] Helleboid P, Heusel M, Duc J et al. The interactome of KRAB zinc finger proteins reveals the evolutionary history of their functional diversification. EMBO J. 2019;38:e101220. 10.15252/embj.2018101220. 31403225 PMC6745500

[49] Imbeault M, Helleboid P-Y, Trono D. KRAB zinc-finger proteins contribute to the evolution of gene regulatory networks. Nature. 2017;543:550–4. 10.1038/nature21683. 28273063

[50] Leung D, Du T, Wagner U et al. Regulation of DNA methylation turnover at LTR retrotransposons and imprinted loci by the histone methyltransferase Setdb1. Proc Natl Acad Sci USA. 2014;111:6690–5. 10.1073/pnas.1322273111. 24757056 PMC4020067

[51] Quenneville S, Turelli P, Bojkowska K et al. The KRAB-ZFP/KAP1 system contributes to the early embryonic establishment of site-specific DNA methylation patterns maintained during development. Cell Rep. 2012;2:766–73. 10.1016/j.celrep.2012.08.043. 23041315 PMC3677399

[52] Padeken J, Methot SP, Gasser SM. Establishment of H3K9-methylated heterochromatin and its functions in tissue differentiation and maintenance. Nat Rev Mol Cell Biol. 2022;23:623–40. 10.1038/s41580-022-00483-w. 35562425 PMC9099300

[53] Ren W, Fan H, Grimm SA et al. Direct readout of heterochromatic H3K9me3 regulates DNMT1-mediated maintenance DNA methylation. Proc Natl Acad Sci USA. 2020;117:18439–47. 10.1073/pnas.2009316117. 32675241 PMC7414182

[54] Yang H, Bai D, Li Y et al. Allele-specific H3K9me3 and DNA methylation co-marked CpG-rich regions serve as potential imprinting control regions in pre-implantation embryo. Nat Cell Biol. 2022;24:783–92. 10.1038/s41556-022-00900-4. 35484247

[55] Zhang T, Termanis A, Özkan B et al. G9a/GLP complex maintains imprinted DNA methylation in embryonic stem cells. Cell Rep. 2016;15:77–85. 10.1016/j.celrep.2016.03.007. 27052169 PMC4826439

[56] SanMiguel JM, Abramowitz LK, Bartolomei MS. Imprinted gene dysregulation in a Tet1 null mouse model is stochastic and variable in the germline and offspring. Development. 2018;145:dev160622. 10.1242/dev.160622. 29530881 PMC5963867

[57] Vasconcelos S, Caniçais C, Chuva de Sousa Lopes SM et al. The role of DNA hydroxymethylation and TET enzymes in placental development and pregnancy outcome. Clin Epigenet. 2023;15:66. 10.1186/s13148-023-01483-z. PMC10127343 37095555

[58] Yamaguchi S, Shen L, Liu Y et al. Role of Tet1 in genomic imprinting erasure. Nature. 2013;504:460–4. 10.1038/nature12805. 24291790 PMC3957231

[59] Amabile A, Migliara A, Capasso P et al. Inheritable silencing of endogenous genes by hit-and-run targeted epigenetic editing. Cell. 2016;167:219–232.e14. 10.1016/j.cell.2016.09.006. 27662090 PMC5039111

[60] O’Geen H, Tomkova M, Combs JA et al. Determinants of heritable gene silencing for KRAB-dCas9 + DNMT3 and Ezh2-dCas9 + DNMT3 hit-and-run epigenome editing. Nucleic Acids Res. 2022;50:3239–53. 35234927 10.1093/nar/gkac123 PMC8989539

[61] O’Geen H, Bates SL, Carter SS et al. Ezh2-dCas9 and KRAB-dCas9 enable engineering of epigenetic memory in a context-dependent manner. Epigenetics Chromatin. 2019;12:26. 31053162 10.1186/s13072-019-0275-8 PMC6498470

[62] Tunnacliffe E, Chubb JR. What is a transcriptional burst?. Trends Genet. 2020;36:288–97. 10.1016/j.tig.2020.01.003. 32035656

[63] Hebenstreit D, Karmakar P. Transcriptional bursting: from fundamentals to novel insights. Biochem. Soc. Trans. 2024;52:1695–702. 10.1042/BST20231286. 39119657 PMC11668302

[64] Zukher I, Dujardin G, Sousa-Luís R et al. Elongation roadblocks mediated by dCas9 across human genes modulate transcription and nascent RNA processing. Nat Struct Mol Biol. 2023;30:1536–48. 10.1038/s41594-023-01090-9. 37783853 PMC10584677

[65] Bernardini A, Hollinger C, Willgenss D et al. Transcription factor IID parks and drives preinitiation complexes at sharp or broad promoters. Trends Biochem Sci. 2023;48:839–48. 10.1016/j.tibs.2023.07.009. 37574371 PMC10529448

[66] Schilbach S, Aibara S, Dienemann C et al. Structure of RNA polymerase II pre-initiation complex at 2.9 Å defines initial DNA opening. Cell. 2021;184:4064–4072.e28. 10.1016/j.cell.2021.05.012. 34133942

[67] Estell C, West S. ZC3H4/restrictor exerts a stranglehold on pervasive transcription. J Mol Biol. 2025;437:168707. 10.1016/j.jmb.2024.168707. 39002716

[68] Scruggs BS, Gilchrist DA, Nechaev S et al. Bidirectional transcription arises from two distinct hubs of transcription factor binding and active chromatin. Mol Cell. 2015;58:1101–12. 10.1016/j.molcel.2015.04.006. 26028540 PMC4475495

[69] Zhang Y, Romanish MT, Mager DL. Distributions of transposable elements reveal hazardous zones in mammalian introns. PLoS Comput Biol. 2011;7:e1002046. 10.1371/journal.pcbi.1002046. 21573203 PMC3088655

[70] Yang P, Wang Y, Macfarlan TS. The role of KRAB-ZFPs in transposable element repression and mammalian evolution. Trends Genet. 2017;33:871–81. 10.1016/j.tig.2017.08.006. 28935117 PMC5659910

[71] Choi JY, Lee YCG. Double-edged sword: the evolutionary consequences of the epigenetic silencing of transposable elements. PLoS Genet. 2020;16:e1008872. 10.1371/journal.pgen.1008872. 32673310 PMC7365398

[72] Bogu GK, Reverter F, Marti-Renom MA et al. Atlas of transcriptionally active transposable elements in human adult tissues. bioRxiv. 2019; 10.1101/714212.

[73] Macchietto MG, Langlois RA, Shen SS. Virus-induced transposable element expression up-regulation in human and mouse host cells. Life Sci. Alliance. 2020;3:e201900536. 10.26508/lsa.201900536. 31964680 PMC6977392

[74] Oomen ME, Rodriguez-Terrones D, Kurome M et al. An atlas of transcription initiation reveals regulatory principles of gene and transposable element expression in early mammalian development. Cell. 2025;188:1156–1174.e20. 10.1016/j.cell.2024.12.013. 39837330

