# Supplementary material for: Transcriptional interference drives intronic polyadenylation at the endogenous H13/Mcts2 locus
Source: Nucleic Acids Res. 2026 Jun 27;54(12):gkag640. doi: 10.1093/nar/gkag640 (PMC13309782; doi:10.1093/nar/gkag640)
Supplement: gkag640_Supplemental_File [file gkag640_supplemental_file.pdf]

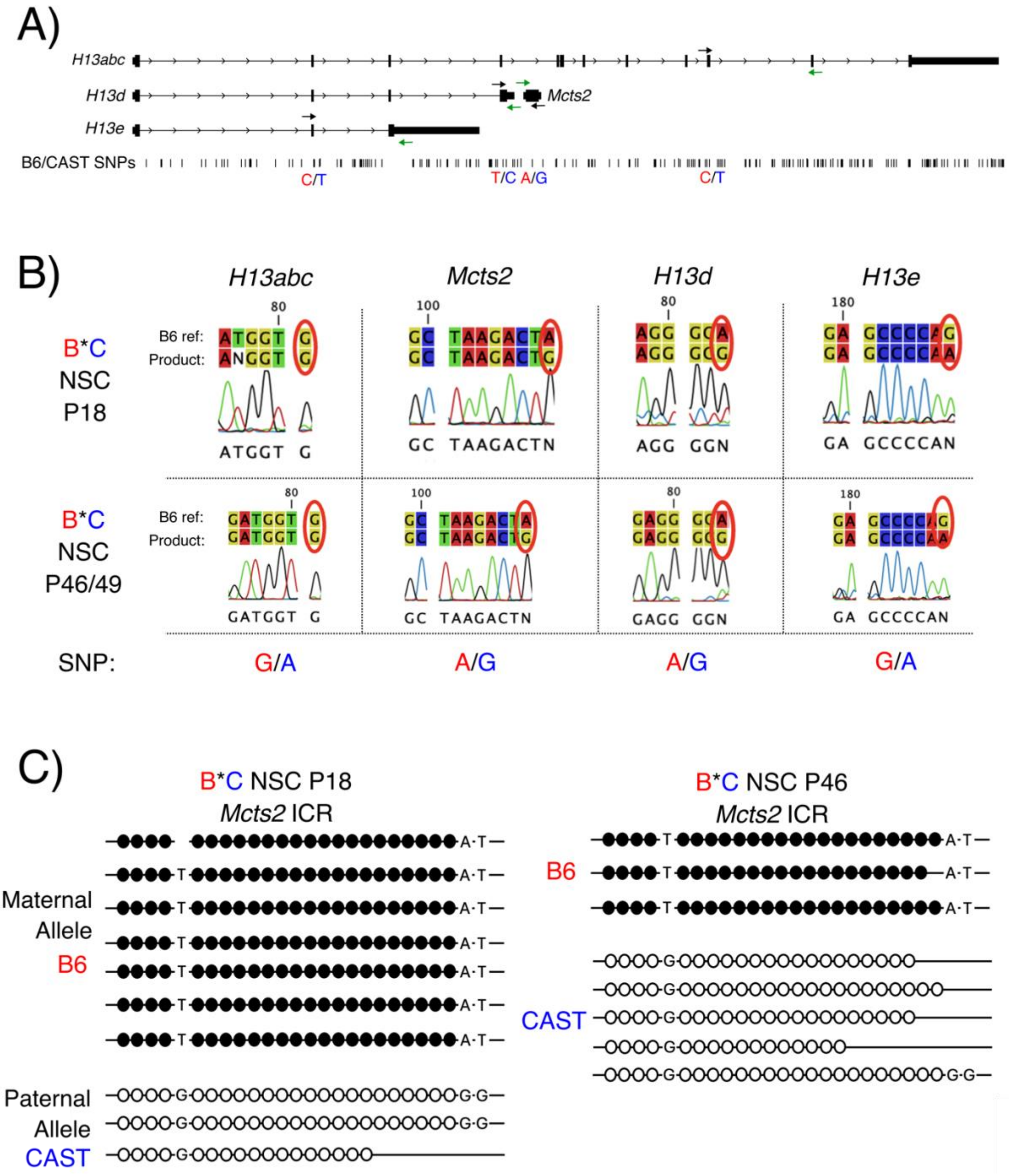

Supplemental Figure 1. B*C Neural Stem cells are a stable epigenetic model to investigate imprinting at late cell passages. A) Schematic of *H13/Mcts2* using RefSeq mm10 gene annotations. qPCR primer locations illustrated by arrows, and green arrows indicate those used for Sanger sequencing. Individual B6/CAST SNPs across the locus shown by vertical black lines **B)** Sanger sequencing traces showing RT-PCR amplicons of transcripts at the *H13/Mcts2* locus. Demonstrating no change in allelic SNPs from early (P18) to late (P46/49) cell passages. **C)** DNA methylation analysis demonstrates no change across both maternal and paternal allele between early and late cell passages at the *Mcts2* ICR.

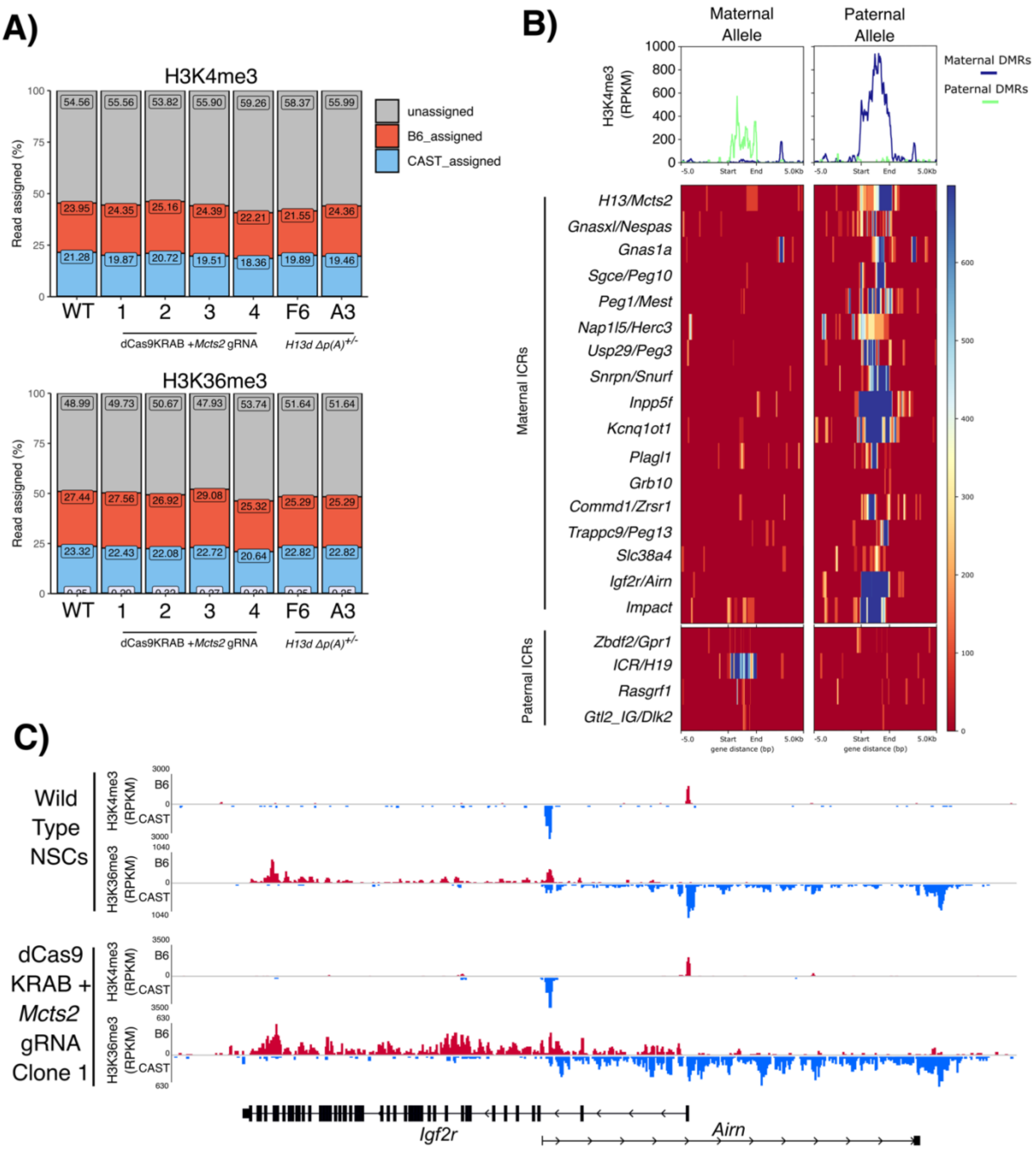

Supplemental Figure 2. Specificity of allelic CUT&RUN in B*C neural stem cells. A) Proportion of H3K4me3 and H3K36me3 CUT&RUN reads that can be assigned to either the maternal (B6) or paternal (CAST) allele, showing that roughly less than a quarter of reads map specifically to either allele in all cell lines examined. **B)** H3K4me3 signal at all the maternal and paternal ICRs. H3K4me3 signal is localised primarily to paternally aligned CAST reads at the region of the maternal ICRs. This is subsequently absent on the maternal allele, as no reads align there likely due to DNA methylation at those ICRs. **C)** Paternal expression of *Airn* and maternal expression of *Igf2r* illustrated by allele-specific CUT&RUN profiles of H3K4me3 at promoters and H3K36me3 mapping elongation across the respective imprinted genes in WT NSCs and Clone 1 of the dCas9:KRAB + *Mcts2* gRNA lines.

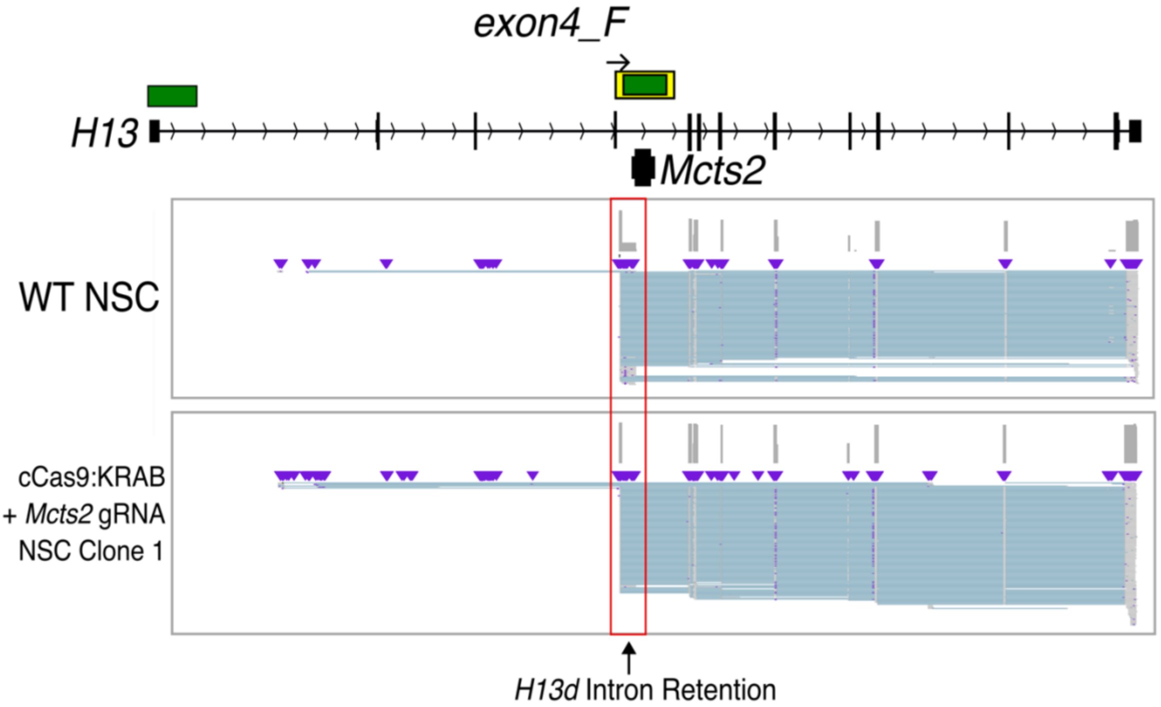

Supplemental Figure 3. Loss of paternal *H13d* intron retention when *Mcts2* is silenced. Long-read sequencing and mapping of 3'RACE products using a forward primer initiation from Exon4 of *H13*. Individual reads are plotted as grey (exons) and blue (introns) lines, indicating loss of *H13d* intron retention events (red box) when *Mcts2* is silenced by dCas9:KRAB and no other impact on locus specific isoforms.

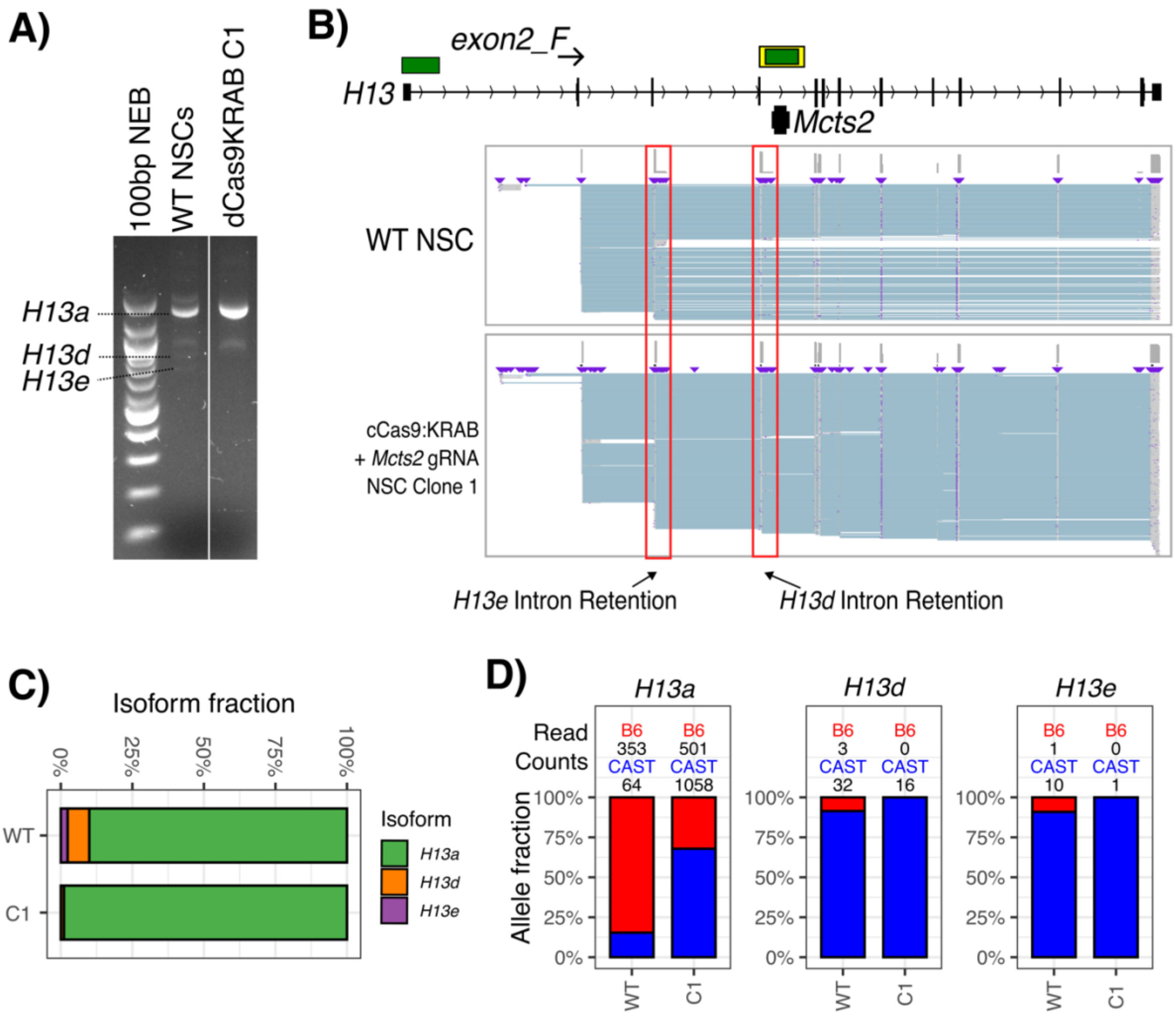

Supplemental Figure 4. Loss of paternal *H13e* intron retention when *Mcts2* is silenced. A) 3'RACE products of *H13* transcripts using a primer initiation from Exon2 capturing, *H13a*, *H13d* and *H13e*. *H13d* and *H13e* are reduced when *Mcts2* is silenced. **B)** Sequencing and mapping of 3'RACE products when *Mcts2* is silenced indicates lack of intronic polyadenylation of *H13d* and *H13e* with a **C)** reduction in their overall transcript proportion levels and **D)** increase in H13a being expressed from the paternal allele and no changes in similar to observations in Figure 3.

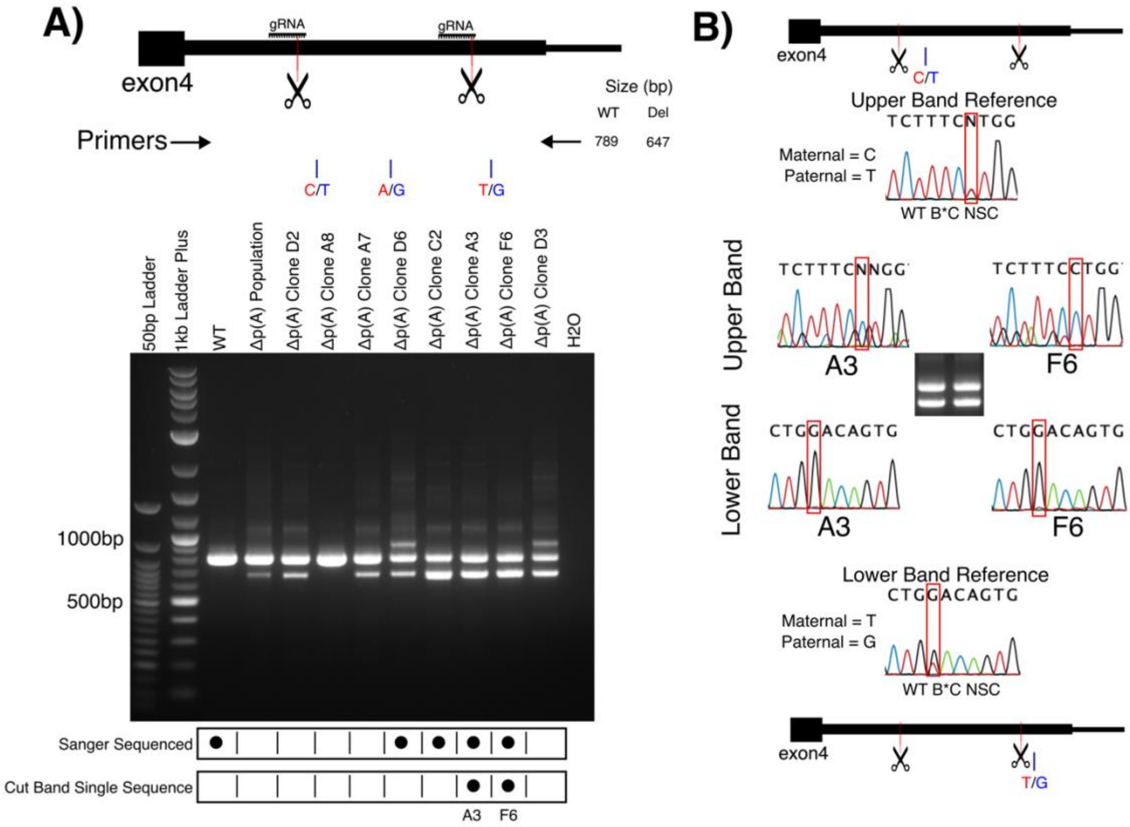

Supplemental Figure 5. Generation of paternal specific *H13d* polyadenylation site deletion cell lines. A) PCR genotyping strategy to detect mono-allelic deletion of target polyadenylation site region. Representative PCR of a selection of candidate NSC clonal cell lines. Further Sanger sequencing revealed two clones exhibit a single sequence in both the upper and lower band, suggestive of a single clonal population. **B)** Sanger sequencing validation of paternal specific *H13d* polyadenylation site knockout. The upper band, of the unedited allele, only demonstrated maternal specific SNPs (Maternal = C, Paternal = T) in both candidate clones A3 and F6. The lower band, edited allele, only demonstrated paternal specific SNPs in both clones (Maternal = T, Paternal = G).

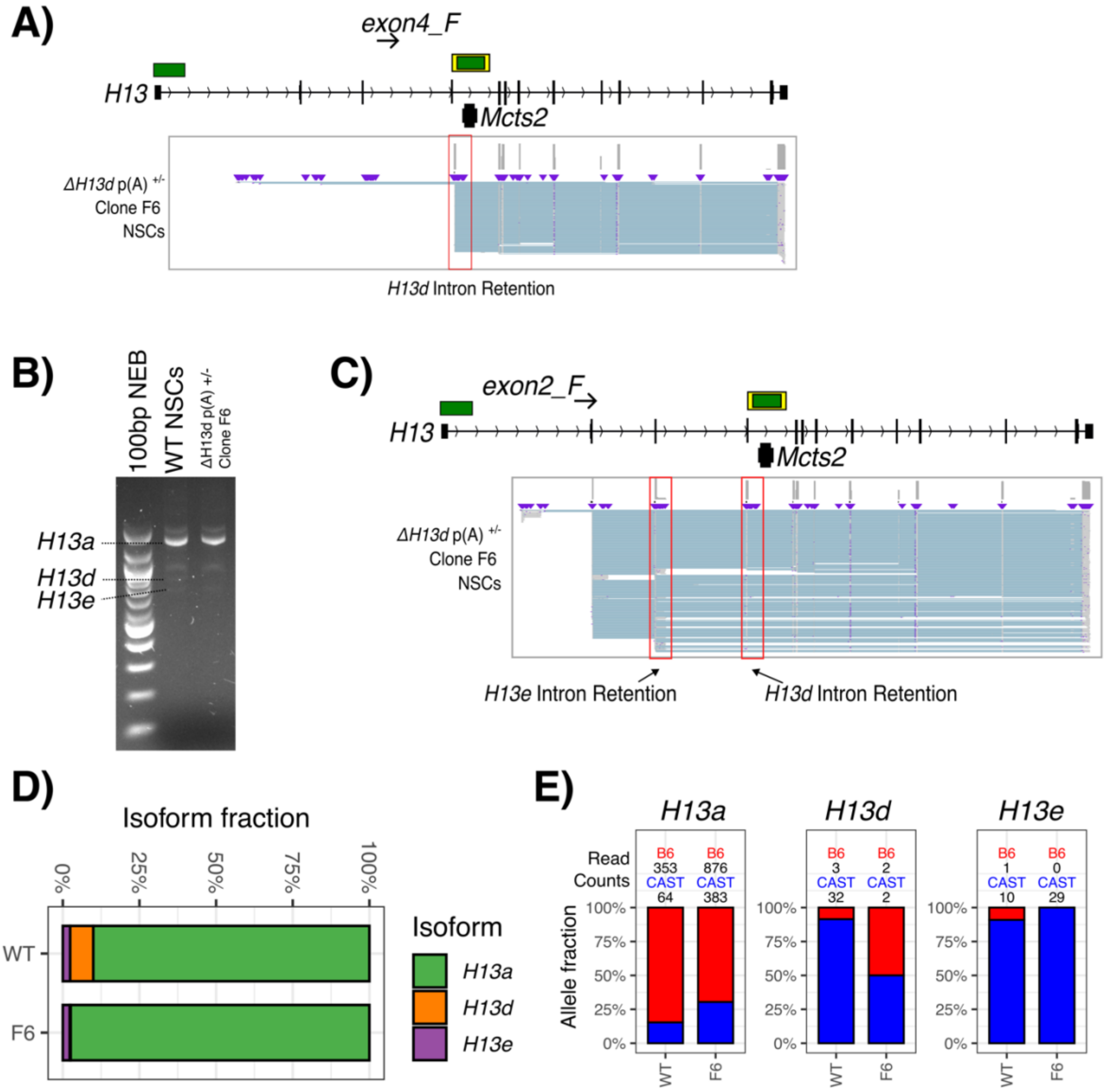

Supplemental Figure 6. Intronic polyadenylation prevention via deletion of *H13d* intronic polyadenylation site does not impact *Mcts2* expression. A) Sequencing and mapping of 3'RACE products shown in Figure 4D showing expected ablation of H13d intronic polyadenylation. **B)** 3'RACE of WT and in *ΔH13d poly(A)*$^{+/-}$ NSC Clone F6 using a forward primer initiation from Exon2 indicates loss of *H13d* without impacting *H13e* indicated also by **C)** sequencing and alignment and, **D)** total *H13* transcript proportion quantification. **E)** When H13d polyadenylation is ablated, *H13abc* is still predominantly expressed from the maternal allele without impacting *H13e* expression.

Use	Target	F/R	Sequence 5' 3'
qPCR	*Mcts2*	Forward	CCCGTCAAAATAGTGAGATGC
		Reverse	GGCAGGATAAACGGGTATTTG
qPCR	*H13abc*	Forward	TGAAGAAGAACACGCACACC
		Reverse	ACAGGAAAGCCGATGCAG
qPCR	*H13d*	Forward	TCAGCTGCTCTTCACACAGG
		Reverse	TGAACCAGGGCCATACAAAT
qPCR	*H13e*	Forward	ATCGCCAGCTGCACACTC
		Reverse	AAAGACCATGTTCCCATCCA
qPCR	*Ppia*	Forward	GTGGTCTTTGGGAAGGTGAA
		Reverse	TTACAGGACATTGCGAGCAG
qPCR	*Rpl30*	Forward	AGTCTCTGGAGTCGATCAACT
		Reverse	AGCCAGTGTGCATACTCTGTAG
qPCR	*Tbp*	Forward	GCGATTTGCTGCAGTCATCA
		Reverse	CAGCTCCCCACCATGTTCTG
DNAm	*Mcts2* ICR	Forward	GGGATGTTTGGGATAGTAAT
		Reverse	TTACCCCACTAATTCTTCTTC
RT-PCR	*H13abc*	Forward	ACATGCCAGAAACCATCACC
		Reverse	ACAGGAAAGCCGATGCAG
Genotyping	*H13d* p(A) KO	Forward	TGTATGGCCCTGGTTCATACCC
		Reverse	TGCGCTGATCCTCACGTGATTA
3'RACE	*H13a* + *H13d* (Exon4)	Forward	GATTACGCCAAGCTTGCCAACTTCCCAAACCGCCAGTATC
		Reverse	Provided by SMARTer® RACE 5'/3' Kit
3'RACE	*H13a* + *H13d* + *H13e* (Exon2)	Forward	GATTACGCCAAGCTTGACATGCCAGAAACCATCACCAGTCG
		Reverse	Provided by SMARTer® RACE 5'/3' Kit
3'RACE (nested)	*H13a* + *H13d* + *H13e* (Exon2)	Forward	TGAAGAAGAACACGCACACC
		Reverse	Provided by SMARTer® RACE 5'/3' Kit

Supplemental Table 1. List of primers used within this study.
